# Spatiotemporal Patterns of the Omicron Wave of COVID-19 in the United States

**DOI:** 10.3390/tropicalmed8070349

**Published:** 2023-06-30

**Authors:** Siyuan Zhang, Liran Liu, Qingxiang Meng, Yixuan Zhang, He Yang, Gang Xu

**Affiliations:** 1 School of Resource and Environmental Sciences, Wuhan University, Wuhan 430079, China; siyuanzhang@whu.edu.cn (S.Z.); zhangyixuan@whu.edu.cn (Y.Z.); xugang@whu.edu.cn (G.X.); 2 School of Remote Sensing and Information Engineering, Wuhan University, Wuhan 430079, China; liranliu@whu.edu.cn; 3 Transportation Development Center of Henan Province, Zhengzhou 450016, China; yanghe8813@hotmail.com

**Keywords:** COVID-19, infectious diseases, spatiotemporal pattern, space-time scan, epicenter

## Abstract

COVID-19 has undergone multiple mutations, with the Omicron variant proving to be highly contagious and rapidly spreading across many countries. The United States was severely hit by the Omicron variant. However, it was still unclear how Omicron transferred across the United States. Here, we collected daily COVID-19 cases and deaths in each county from 1 December 2021 to 28 February 2022 as the Omicron wave. We adopted space-time scan statistics, the Hoover index, and trajectories of the epicenter to quantify spatiotemporal patterns of the Omicron wave of COVID-19. The results showed that the highest and earliest cluster was located in the Northeast. The Hoover index for both cases and deaths exhibited phases of rapid decline, slow decline, and relative stability, indicating a rapid spread of the Omicron wave across the country. The Hoover index for deaths was consistently higher than that for cases. The epicenter of cases and deaths shifted from the west to the east, then southwest. Nevertheless, cases were more widespread than deaths, with a lag in mortality data. This study uncovers the spatiotemporal patterns of Omicron transmission in the United States, and its underlying mechanisms deserve further exploration.

## 1. Introduction

The COVID-19 epidemic caused by the severe acute respiratory syndrome coronavirus 2 (SARS-CoV-2) has brought unprecedented changes to the world. As an RNA virus, SARS-CoV-2 is known for its high mutation rate, which results in constant changes during transmission across populations. Up to now, five variants of COVID-19 have been widely reported: B.1.1.7 (Alpha), B.1.351 (Beta), P.1 (Gamma), B.1.617.2 (Delta), and B.1.1.529 (Omicron) [1]. The Omicron variant, discovered in South Africa in November 2021, has been found to weaken natural and vaccine immunity significantly compared to previous variants [2,3]. It is highly contagious and has rapidly spread to many countries [4]. The United States was also severely hit by Omicron at the end of 2021 [5].

Spatial analysis is indispensable to uncover spatiotemporal patterns of infectious disease transmission. Numerous studies on COVID-19 using spatial analysis and GIS technologies can be divided into three aspects. Firstly, real-time maps based on different themes, such as the spatial distribution of cases, risk zones, vaccination status, etc. [6]. Secondly, a series of spatial analysis methods, such as kernel density estimation, spatial autocorrelation analysis [7,8], and space-time scan statistics were used to quantitatively identify the clustering patterns of COVID-19 [9,10,11]. The most obvious feature is that cities with high population density and social interaction are found to have higher clustering of cases [12,13]. Thirdly, environmental (such as temperature and humidity) and socioeconomic factors (economic status and demographics) are found to influence the epidemic [14]. The restriction of human mobility can effectively slow down the transmission of the COVID-19 epidemic [15,16]. For example, in early 2020, the destination and size of population movements before the closure of Wuhan were found to determine the final spatial distribution of COVID-19 cases in China [17].

In the United States, since COVID-19 initial outbreak in early 2020, the spread of the epidemic has continued to influence the nation’s social, economic, and healthcare landscapes. The disease has demonstrated several distinct characteristics. Firstly, the spread of the epidemic varies geographically, with regions such as New York State being at the center of the epidemic’s spatially shifting network [18]. These areas have a high basic transmission number R_0_ value [19], a significantly larger hotspot size than cold-spot size [20], and a high risk of epidemic transmission. Long-distance, high-intensity transmission is dominant between network centers, while other areas are dominated by short-range, adjacent transmission [18]. Second, the development of COVID-19 varies over time. Troy McMahon [21] constructed a value C(r) for describing spatial correlation, which can classify epidemics into three phases: localized, dormant, and system-wide outbreak according to their value magnitude. Non-pharmaceutical interventions (NPIs) are effective in controlling epidemics. Studies have shown a significant positive correlation between personnel contact and epidemic severity using social index analysis [22,23]. Additionally, the transmission of COVID-19 in the United States has been characterized by fluctuations, with periods of relative containment followed by resurgences, and is still experiencing recurrences now. These waves have exposed the vulnerabilities in the country’s public health infrastructure, leading to far-reaching consequences for individuals, communities, and the whole nation. The transmission dynamics of COVID-19 in the United States have been influenced by a complex interplay of factors. Early efforts were made to implement preventive measures, such as lockdowns, mask mandates, and widespread testing. However, the virus managed to find its way to transmission due to inconsistent adherence to guidelines, the emergence of new variants, and vaccine hesitancy [24,25,26].

The characteristics of COVID-19’s spread varied noticeably at different phases. The most contagious subvariant of Omicron is the BA.5 strain, which is also the dominant subvariant globally. The patterns of spread also varied across different states and regions, with densely populated cities experiencing higher infection rates and increased strain on healthcare systems. However, it was still unclear how Omicron transferred across the United States and how its transmission characteristics differ from previous outbreaks. Further investigation is necessary. Revealing the spatiotemporal transmission mode and characteristics of the COVID-19 epidemic from the perspective of GIS is of great significance in controlling the transmission of a new wave of epidemics triggered by virus mutations in the future.

We collected daily COVID-19 cases and deaths in each county to quantify spatiotemporal patterns of the Omicron wave (from 1 December 2021 to 28 February 2022) of COVID-19. This paper is structured as follows: Section 2 presents the study area and data used in the research and introduces the main methods. Section 3 outlines the results of space-time scan statistics, the Hoover index analysis, and trajectories of the epicenter. Section 4 summarizes contributions and indicates future works. Finally, Section 5 draws conclusions.

## 2. Materials and Methods

### 2.1. Time Series COVID-19 Data

Our study area is the continental United States, except Hawaii and Alaska. The time-series COVID-19 cases and deaths at the county level were collected from the USAFacts website (https://usafacts.org/, accessed on 1 March 2023). There are over 3000 counties in the continental United States.

### 2.2. Space-Time Scan Statistic

Space-time scan analysis is a widely used method for disease surveillance due to its ability to identify spatiotemporal clusters with elevated disease risk [27,28]. The spatiotemporal scan statistic utilizes a changing cylindrical window that changes shape to scan the entire study area. The bottom of the cylinder represents the potential spatiotemporal cluster, and the height indicates the duration of the potential cluster. The statistics are constructed by moving the center of the scan window, expanding the scan radius, and increasing the scan time range to identify the spatiotemporal clusters.

Assuming that COVID-19 cases follow a Poisson distribution, the likelihood ratio of the observed COVID-19 cases (deaths) in the study area is constant under the null hypothesis $H_{0}$. Under the Poisson model, the expected number of COVID-19 cases (deaths) in each county is proportional to its total population. Then, the expected number of cases *E* meets:

(1) $$E=\overset{n}{\underset{i=1}{\sum }}e_{i}=\overset{n}{\underset{i=1}{\sum }}p_{i}\times \frac{N}{P}$$

where $e_{i}$ and $p_{i}$ are the expected number of cases and population of city i, and *P* and *N* are the total populations and the total number of cases.

The actual number of cases observed in some scan windows is higher than the expected number of cases. Those cylindrical scanning windows can be identified by calculating the log-likelihood ratio (*LLR*). The *LLR* measures the degree of deviation between the actual and expected cases in a given scan window. The larger the *LLR*, the more likely the cylindrical window is to be regarded as a spatiotemporal cluster. The specific calculation formula for calculating the *LLR* is as follows:

(2) $$LLR=\frac{{\left(\frac{n}{e}\right)}^{n}{\left(\frac{N-n}{N-e}\right)}^{N-n}}{{\left(\frac{N}{E}\right)}^{N}}$$

where n and e are the actual number and expected number of cases in the scan window, *N* and *E* are the total actual and expected number of cases in the study area.

To compare the possibility of COVID-19 infection in each cluster, the relative risk (*RR*) was introduced. A higher *RR* value indicates a greater likelihood of COVID-19 infection in the population of the cluster. The calculation formula is as follows:

(3) $$RR=\frac{n/e}{\left(N-n\right)/\left(N-e\right)}$$

The meanings of *N*, n, and e in Equation (3) are the same as those in Equation (2).

### 2.3. The Hoover Index

The spread of COVID-19 is closely associated with human-to-human contact, and research has demonstrated a positive correlation between the number of COVID-19 cases and population size [29,30]. Therefore, as the COVID-19 epidemic continues to spread in the United States, the distribution of cases in each county should converge to the population distribution, and the time taken for this convergence can be used to measure the rate of COVID-19 transmission. The localized Hoover index (HI) is used to measure the similarity of the spatial distribution of the two variables within a given geographic area. To further quantitatively characterize the transmission process of the Omicron wave in the United States, we used the Hoover index to measure the heterogeneity of the distribution of COVID-19 cases and the population distribution in counties, calculated as follows:

(4) $$Hoover_{t}=50\sum_{i=1}^{n}\left|\frac{p_{it}}{\sum_{i=1}^{n}p_{it}}-\frac{n_{it}}{\sum_{i=1}^{n}n_{it}}\right|$$

where $Hoover_{t}$ indicates the Hoover index of the number of COVID-19 cases at time t, ranging from 0 to 100. Larger values indicate greater differences between case distribution and population distribution, with cases concentrated in a few cities and stronger spatial heterogeneity; $p_{it}$ and $n_{it}$ indicate the population and the number of cases in city i at time t, respectively.

### 2.4. Epicenter of COVID-19

The migration trajectory of the epidemic center of gravity can reflect the spatiotemporal transmission trend of the epidemic in a certain space. The longitude and latitude of the epidemic center of gravity in the $i^{th}$ state of the US are calculated as follows:

(5) $$EC\_lon_{i}=\frac{1}{n_{i}}\sum_{j=1}^{n_{i}}C_{ij}*GC\_lon_{ij}$$

(6) $$EC\_lat_{i}=\frac{1}{n_{i}}\sum_{j=1}^{n_{i}}C_{ij}*GC\_lat_{ij}$$

where $n_{i}$ indicates the total number of counties in the $i^{th}$ state, $C_{ij}$ indicates the number of cases/deaths of the $j^{th}$ county in the $i^{th}$ state, $GC\_lon_{ij}$ and $GC\_lat_{ij}$ represent the longitude and latitude of the geographic center of the $i^{th}$ state and the $j^{th}$ county. $EC\_lon_{i}$ and $EC\_lat_{i}$ represent the calculated longitude and latitude of the weighted geographic center of the state. The epidemic center of gravity of the country is calculated the same way as that of the state.

## 3. Results

### 3.1. Spatiotemporal Variabilities of the COVID-19 Epidemic

**Figure 1:**
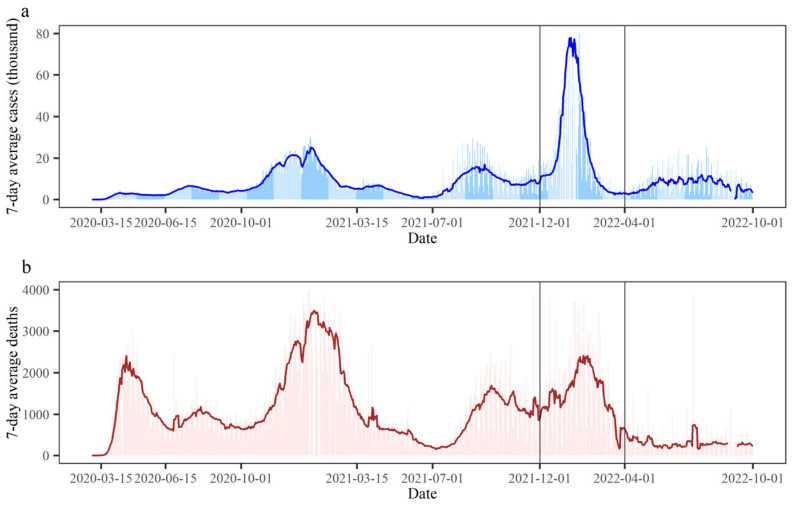
Temporal evolution of the COVID-19 epidemic variations in the United States. (**a**) Newly added COVID-19 cases; (**b**) newly added COVID-19 deaths. The period from 1 December 2021 to 28 February 2022 was defined as the Omicron wave of the COVID-19 epidemic.

The United States witnessed multiple waves of the COVID-19 epidemic, with the most severe one occurring from 1 December 2021 to 28 February 2022, since its initial outbreak. The extremely contagious Omicron mutant strain was the primary contributor to this epidemic wave. In total, there were about 30 million cases, and 170,000 people died as a result. The weekly moving average and daily counts of COVID-19 cases and deaths are shown in Figure 1. The number of new cases in a single day peaked in mid-January 2022, reaching a maximum of more than 1.25 million, whereas the number of new deaths in a single day fluctuated relatively little, reaching a maximum of 4000.

The spatial evolution of cases and deaths in the United States during the transmission phase of the Omicron wave (1 December 2021 to 28 February 2022) are shown in Supplementary Figures S1 and S2. Despite the widespread transmission of COVID-19 throughout the country, the US government did not implement a stringent dynamic zero-COVID policy. Therefore, only Florida, Washington, Wyoming, and a few central counties in the United States had not yet reported cases at the start of the Omicron wave. By 28 February 2022, 99% of counties had reported Omicron cases. While the spatial distribution of deaths followed a similar pattern to that of cases, there were still some differences. The overall trend was moving from the east and west coasts to the center of the nation. Notably, major cities on the east and west coasts, including New York, Los Angeles, Chicago, and Detroit, were significantly impacted during this period.

**Figure 2:**
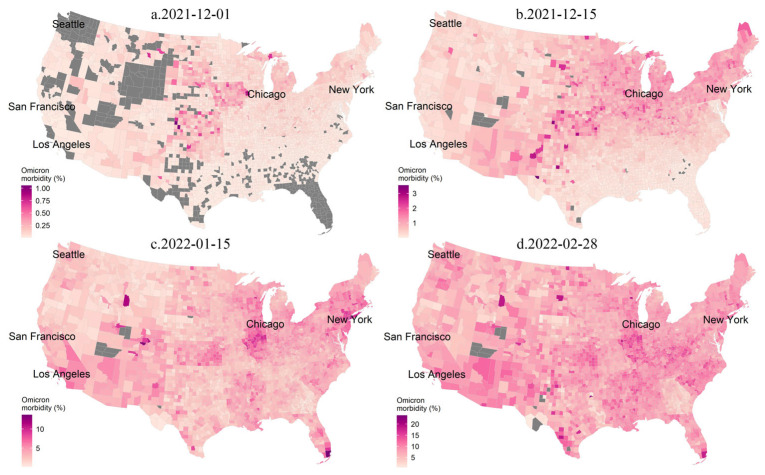
Spatial distributions of the accumulative COVID-19 morbidity during the Omicron wave across the continental United States. The Omicron morbidity is defined as the accumulative COVID-19 cases to the population from November 30, 2021 to a following date. (**a**) 1 December 2021; (**b**) 15 December 2021; (**c**) 15 January 2022; (**d**) 28 February 2022.

The spatial distribution of the morbidity (cumulative number of confirmed cases/total population) and mortality (cumulative number of deaths/total population) on different dates led by the Omicron wave (1 December 2021 to 28 February 2022) are shown in Figure 2 and Figure S3. The spatiotemporal evolution of morbidity and mortality significantly differed from that of cases and deaths. In the early stage of the Omicron wave (1 December 2021 to 15 December 2021), the highest morbidity shifted from the Midwest to the northeastern coastal regions, while the highest mortality was primarily found in the Northwest. The spatial clusters with relatively high mortality were located in the southern and north-central United States.

### 3.2. Space-Time Scan Analysis

**Figure 3:**
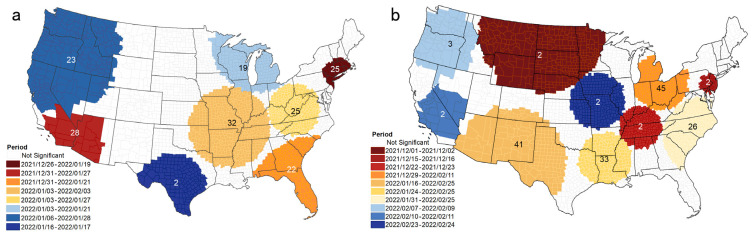
Spatial clusters of COVID-19 cases and deaths during the Omicron wave. (**a**) New cases; (**b**) new deaths. The numbers in clusters indicate days of duration. Color in the clusters and the figure legend are the same, and the color gradient (dark red to dark blue) indicates the temporal change based on the initial date of the cluster. The cluster numbers in Table 1 and Table S1 indicate the rank of the relative risk for each cluster.

Statistically significant spatiotemporal clusters of daily Omicron cases and deaths by county between 1 December 2021 and 28 February 2022 are shown in Figure 3. The highest and earliest cluster, spanning from 26 December 2021 to 19 January 2022, is located near the border of the state of New York and Massachusetts, encompassing 45 counties with a relative risk greater than 1, indicating a higher risk of Omicron transmission in this area compared to other regions of the country. Among these, Providence County had an RR of 1.67, with 95,245 cumulative cases noted, while Richmond County and New York County, located in the New York Metropolitan Area, had RRs of 1.66 and 1.56, with 70,649 and 225,76 cases. From 31 December 2021 to 27 January 2022, the second-highest cluster occurred at the Arizona–Californian interface, covering a part of San Francisco. Parts of Florida, Alabama, Georgia, and South Carolina formed clusters of cases from 31 December 2021 to 21 January 2022, including 43 counties with RR > 1. Miami-Dade County, Broward County, and Palm Beach County, all in Miami, had RRs of 2.04, 1.35, and 1.01. The cluster with the highest relative risk was in South Texas, where the RR of Loving County was the highest, reaching 10.35. This high relative risk can be attributed to the county’s small population, resulting in a small expected number of cases.

### 3.3. Hoover Index Analysis

**Figure 4:**
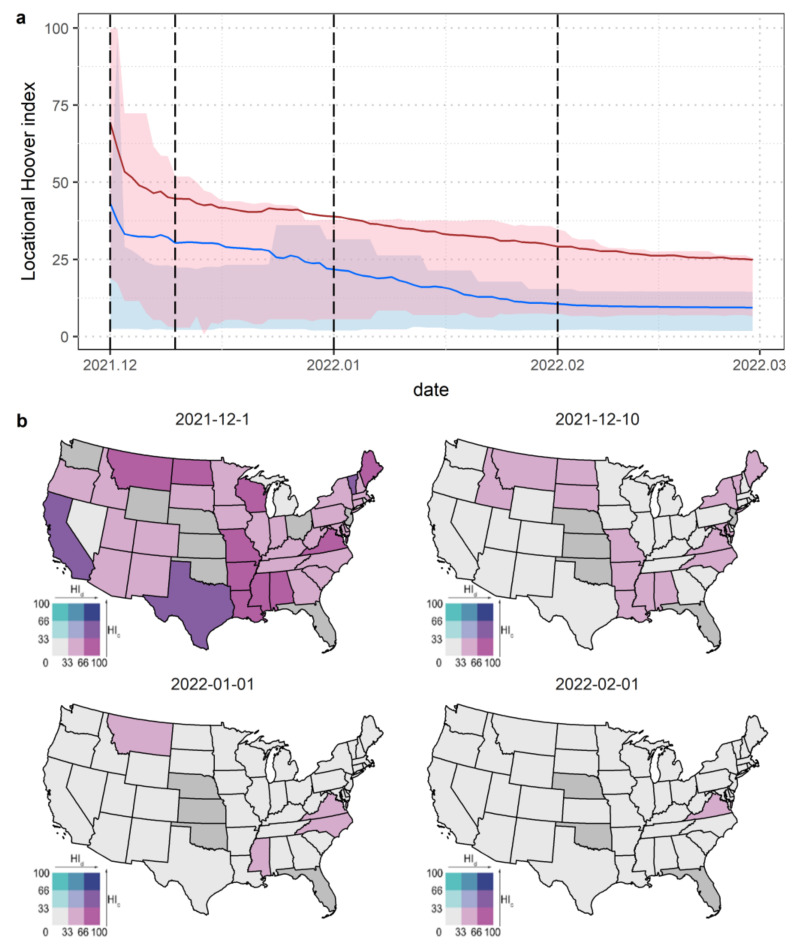
Spatiotemporal evolutions of the Omicron wave of COVID-19 revealed by the Hoover index. (**a**) Temporal variations of locational Hoover index from 1 December 2021 to 28 February 2022 in the United States. (**b**) Bivariate choropleth map of locational Hoover index for COVID-19 cases and deaths at the state level on four representative days.

The trend of HI values in the United States during the Omicron wave is shown in Figure 4a. HI values close to 100 indicate concentration in a few cities, while HI values close to 0 indicate a more homogeneous spread. The blue and red curves represent the HI for cases and deaths, while the blue and red areas surrounding the curves represent the range of HI values on a state scale. Both the HI for cases and deaths experienced a rapid decline (1 December 2021 to 10 December 2021), followed by a slow decline (10 December 2021 to 1 February 2022) and a plateau phase (1 February 2022 to 28 February 2022), stabilizing at around 9.7 and 25.4 in February 2022. Compared to the HI for cases, which was at a low level at the beginning of the wave (HI < 50), the HI for deaths fell below 50 over 4 days, indicating that the Omicron epidemic spread widely across the country in a very short period. The HI for deaths was consistently higher than the HI for cases, indicating that the high level of healthcare in the United States helped avoid the collapse of the healthcare system due to a public health crisis.

The epidemic spread across states with significant spatial heterogeneity, as shown in Figure 4b, which plots the bivariate choropleth map of the HI for cases and deaths at the state scale, where the gray color indicates that the state has not yet reported cases/deaths or cases/deaths have not yet been assigned to counties. Overall, the Omicron wave was spreading very rapidly within the United States. As of 10 December 2021, the HI for cases had all dropped below 33, and the HI for deaths had also dropped below 50, except for Virginia and Mississippi. The states with the widest distribution of cases and deaths were Rhode Island (HI cases = 2.15) and Delaware (HI deaths = 2.84), both located in the eastern coastal areas. Notably, in Wyoming, which is located in the center of the United States, deaths spread more quickly than cases between 1 December 2021 and 4 December 2021. This corresponds to Figure 3, where Wyoming was included in the area of death clustering that formed between 1 December and 2 December.

### 3.4. Spatial Transformation of the Epicenter

**Figure 5:**
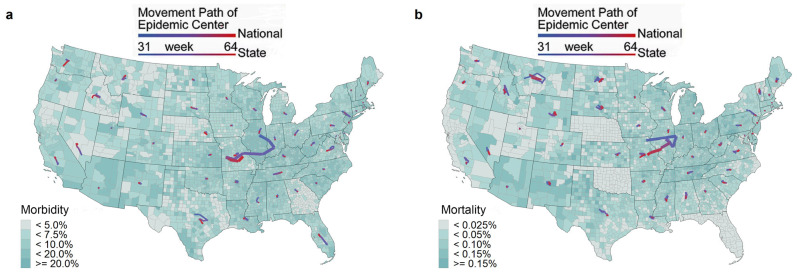
Spatial transformation of the epicenter of the Omicron wave of COVID-19 across the continental United States. (**a**) Epicenter of cases. (**b**) Epicenter of deaths.

The Omicron wave was highly contagious. We calculated the geographical centers of the epidemic and used their migration trajectories to examine the spatial transformation of cases and deaths in the Omicron wave in the United States, as shown in Figure 5. Nationally, both the epicenter of cases and deaths shifted from west to east, then to the southwest, passing through three states before ending in Missouri, indicating a turning point in the epidemic. The eastern part of the country was more severely affected before the turning point, while the southwestern part of the country developed more rapidly thereafter. Cases were more widespread than deaths and tended to move eastward again, indicating a smaller increase in death data and a certain lag. At the state scale, most states had a limited range of movement of the center of gravity of Omicron. However, states with a wider range tended to move vertically towards the epidemic’s center of gravity, either southward or northward. Eventually, the trajectory of deaths moved closer to the coast while the trajectory of cases in the southeastern states moved closer to Tennessee.

## 4. Discussion

This study measures and compares the patterns of transmission of Omicron cases and deaths among US counties at spatial and temporal scales. Spatially, the distribution patterns of cases and deaths differed, but the overall trend shifted from the east and west coasts to the national center. The spatial evolution of morbidity and mortality differed significantly from that of cases and deaths, with the highest morbidity shifting from the Midwest to the Northeast during the initial phase of the Omicron wave, while the highest mortality occurred mainly in the Northwest. The highest and earliest cluster was located in the Northeast. Temporally, the Hoover indexes of both cases and deaths underwent a rapid decline, a slow decline, and a relatively stable phase. The Hoover indexes for deaths were consistently higher than those for cases, indicating that the Omicron rapidly spread across the country in a very short period. The epicenter of cases and deaths shifted from the west to the east and then to the southwest, passing through three states and ending in Missouri. However, cases were more widespread than deaths and tended to move eastward again, indicating a smaller increase in mortality and a certain lag.

A study conducted in England reported that spatial growth of the Omicron variant was 2.81 times faster than that of the Delta variant and 3.76 times faster than that of the Alpha variant [31]. The faster transmission of Omicron was widely found in other countries [32,33]. Our results confirm the highly contagious nature of Omicron and demonstrate its rapid spread in the United States. The clustering and spread of Omicron were different from that of early COVID-19 during the first half of 2020. Areas of high relative risk (RR) for COVID-19 were predominantly in New England, the Southeast, and the Southwest in the first COVID-19 wave [34]. But the highest and earliest cluster of Omicron wave was located in the Northeast. The clustering and spread of Omicron in each state also had different patterns, suggesting that the spread of the virus in each US state cannot be explained by a single factor but was influenced by a combination of complex factors. Firstly, the United States is large and unequal, with differences in the quantity and quality of health resources (e.g., hospital beds and physicians) and income. Secondly, certain cities during Omicron wave failed to receive timely testing or lacked well-structured genomic surveillance results due to variations in testing access, differential diagnostic capacity, and inconsistent certification [35,36]. Thirdly, the policies adopted by states in response to the epidemic and the timing of policy implementation varied [37,38].

To prevent the rapid spread of the virus and ensure consistent resource allocation, the government should implement effective policies (e.g., physical isolation measures) to control the spread of the outbreak [39,40,41]. Our findings suggest that in the United States, Omicron deaths spread slower than cases, indicating the successful implementation of containment policies and the resilience of the US healthcare system [42]. However, some states like Rhode Island failed to control Omicron spread effectively, while Delaware’s healthcare system struggled to reduce Omicron mortality, and Wyoming faced recurrent outbreaks. If timely action is not taken, this could foreshadow similar outcomes in other parts of the United States. Failure to avoid this new round of transmission will facilitate the emergence of new variants of concern, posing a threat to global health security and leading to a completely avoidable humanitarian crisis. Furthermore, targeted government containment measures and coordinated epidemiological and genomic surveillance are needed during infectious disease outbreaks to prevent further loss of life resembling that with COVID-19 [13,42,43].

This study also has limitations. First, we used the space-time scan statistic, Hoover index, and epicenter of COVID-19 models to simulate the spread of Omicron across the United States without accounting for multiple complexities such as differences in health resource quantity and quality, and a dense urban network that connects municipalities through transportation, services, and businesses. There are already studies integrating other elements of modeling to study COVID-19, and we still have to keep improving the model in the future [44]. Second, although the rate of transmission of Omicron deaths was slower than that of cases in most states, there was variation in outbreaks in some states such as Rhode Island, Delaware, and Wyoming, and we did not look deeply into individual state influences. In the future, we need to integrate multiple factors such as outbreak control measures and sanitary conditions to model the spatiotemporal evolution of COVID-19. Additionally, further research is necessary to generalize these patterns to countries with different levels of development and local contexts and identify the underlying causes of these differences.

## 5. Conclusions

This study examines the spatial and temporal patterns of Omicron transmission of cases and deaths across counties in the United States. Overall, Omicron cases and deaths shifted from the east and west coasts to the center of the country. The highest and earliest cluster was located in the Northeast. The Hoover indexes of both cases and deaths underwent a rapid decline, a slow decline, and a relatively stable phase, indicating that the Omicron epidemic spread widely across the country within a short period. In contrast, the Hoover index of deaths was consistently higher than that of cases, indicating that the high level of healthcare in the United States has helped to prevent the collapse of the healthcare system. The epicenter of cases and deaths shifted from the west to the east, then southwest through three states before ending in Missouri. However, cases were more widespread than deaths, and there was a lag in mortality data.

The COVID-19 epidemic nearly ended, but we will face the threat of other emerging infectious diseases. In the prevention and control of future outbreaks, it is crucial for governments to take immediate and rational measures. First, healthcare resources must be enhanced to effectively control mortality while also improving the resilience of the healthcare system to prevent their collapse due to large-scale outbreaks. Second, governments should take containment measures such as lockdowns and accelerate vaccinations to prevent the spread of outbreaks. Furthermore, it is essential to implement coordinated epidemiological and genomic surveillance to monitor the emergence of novel variants and prevent threats to global health security.

## Figures and Tables

**Table 1:** Spatiotemporal cluster information of new Omicron cases in the US at different phases.

Cluster	Duration (Days)	Number of Counties	*p*	Observed	Expected	RR	Number of Counties with RR > 1
1	26 December 2021–19 January 2022	65	<0.001	2,449,866	806,725	3.22	45
2	31 December 2021–27 January 2022	20	<0.001	2,316,904	897,684	2.72	6
3	31 December 2021–21 January 2022	246	<0.001	1,821,212	684,190	2.77	43
4	3 January 2022–3 February 2022	644	<0.001	2,209,112	1,034,411	2.23	341
5	3 January 2022–27 January 2022	382	<0.001	1,726,603	806,957	2.21	217
6	3 January 2022–21 January 2022	239	<0.001	1,326,786	594,240	2.29	83
7	6 January 2022–28 January 2022	218	<0.001	1,570,202	742,166	2.18	28
8	16 January 2022–17 January 2022	150	<0.001	287,673	38,676	7.5	29

## Data Availability

The time-series COVID-19 cases and deaths at the county level are publicly available at the USAFacts website (https://usafacts.org/, accessed on 1 March 2023).

## Supplementary Materials

The following supporting information can be downloaded at: https://www.mdpi.com/article/10.3390/tropicalmed8070349/s1, Figure S1: Spatial distributions of COVID-19 cases during the Omicron wave across the continental United States; Figure S2: Spatial distributions of COVID-19 deaths during the Omicron wave across the continental United States; Figure S3: Spatial distributions of COVID-19 mortality during the Omicron wave across the continental United States.; Table S1: Spatiotemporal cluster information of new COVID-19 deaths in the US at different phases.
