# Supplementary material for: Spatiotemporal Patterns of the Omicron Wave of COVID-19 in the United States
Source: Trop Med Infect Dis. 2023 Jun 30;8(7):349. doi: 10.3390/tropicalmed8070349 (PMC10385263; doi:10.3390/tropicalmed8070349)
Supplement: Supplementary file 1 [file tropicalmed-08-00349-s001.zip › tropicalmed-2424988-supplementary.pdf]

Supplementary Materials

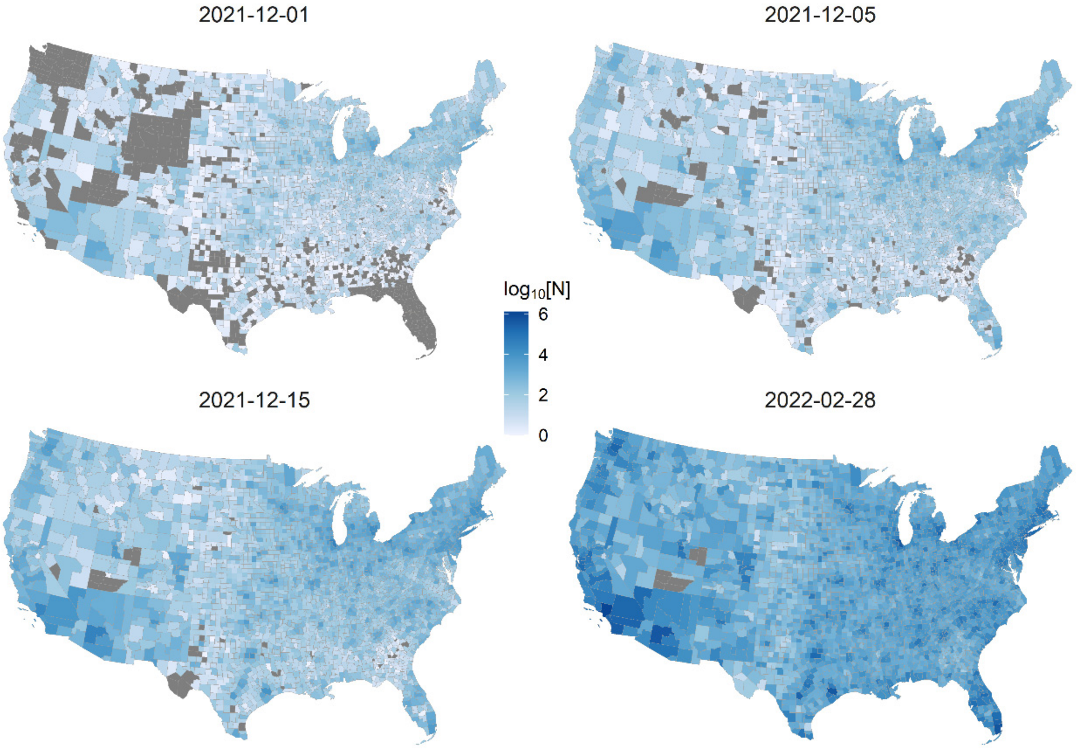

Figure S1. Spatial distributions of the COVID-19 cases during the Omicron wave across the continental United States. The Omicron cases is defined as the accumulative COVID-19 cases from November 30, 2021 to a following date. (a) 1 December 2021; (b) 5 December 2021; (c) 15 December 2021; (d) 28 February 2022.

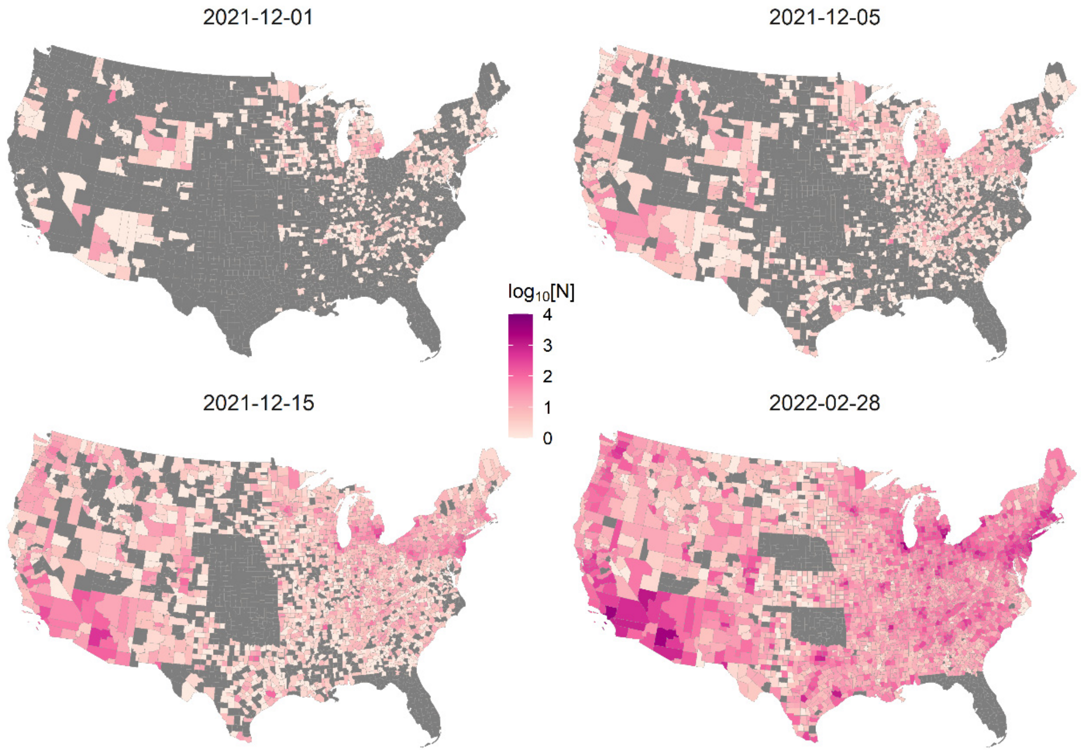

Figure S2. Spatial distributions of the COVID-19 deaths during the Omicron wave across the continental United States. The Omicron deaths is defined as the accumulative COVID-19 deaths from November 30, 2021 to a following date. (a) 1 December 2021; (b) 5 December 2021; (c) 15 December 2021; (d) 28 February 2022.

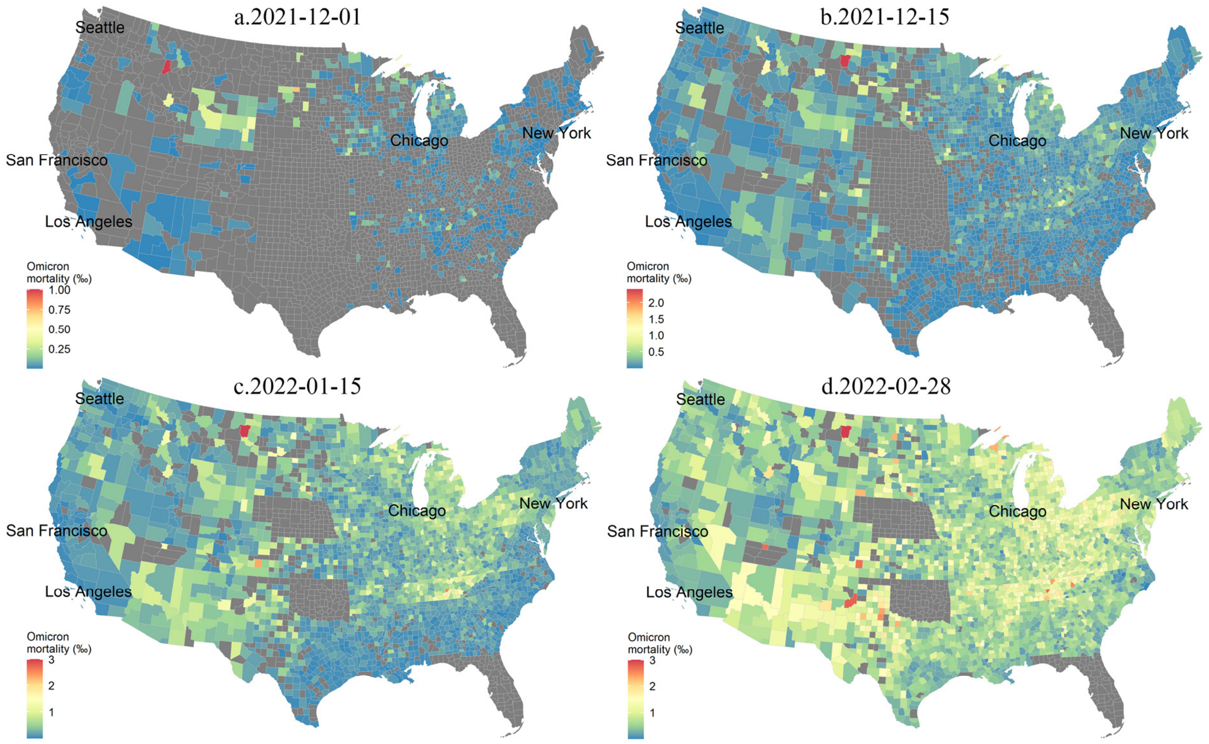

Figure S3. Spatial distributions of the COVID-19 mortality during the Omicron wave across the continental United States. The Omicron mortality is defined as the accumulative COVID-19 deaths to the population from November 30, 2021 to a following date. (a) 1 December 2021; (b) 15 De-cember 2021; (c) 15 January 2022; (d) 28 February 2022.

Table S1. Spatiotemporal cluster information of newly added COVID-19 deaths in the US at different phases.

Cluster	Duration(days)	Number of counties	p	Observed	Expected	RR	Number of counties with RR > 1
1	1 December 2021—2 December 2021	321	<0.001	300	81	3.7	220
2	15 December 2021—16 December 2021	44	<0.001	2057	179	11.61	39
3	22 December 2021—23 December 2021	234	<0.001	1885	132	14.45	204
4	29 December 2021—11 February 2022	286	<0.001	15659	7565	2.19	270
5	16 January 2022—25 February 2022	202	<0.001	5334	2825	1.92	155
6	24 January 2022—25 February 2022	222	<0.001	3345	1766	1.91	146
7	31 January 2022—25 February 2022	362	<0.001	6595	3598	1.87	221
8	7 February 2022—9 February 2022	94	<0.001	391	208	1.88	35
9	10 February 2022—11 February 2022	26	<0.001	788	264	3	7
10	23 February 2022—24 February 2022	297	<0.001	716	148	4.87	191
